# Reduction of Dietary Fat Rescues High-Fat Diet-Induced Depressive Phenotypes and the Associated Hippocampal Astrocytic Deficits in Mice

**DOI:** 10.3390/metabo15070485

**Published:** 2025-07-18

**Authors:** Kai-Pi Cheng, Hsin-Hao Chao, Chin-Ju Hsu, Sheng-Feng Tsai, Yen-Ju Chiu, Yu-Min Kuo, Yun-Wen Chen

**Affiliations:** 1 Department of Internal Medicine, National Cheng Kung University Hospital, College of Medicine, National Cheng Kung University, Tainan 70101, Taiwan; 2 Department of Psychiatry, Ditmanson Medical Foundation Chiayi Christian Hospital, Chiayi 600566, Taiwan; 3 Department of Pharmacology, College of Medicine, National Cheng Kung University, Tainan 70101, Taiwan; 4 Institute of Basic Medical Sciences, College of Medicine, National Cheng Kung University, Tainan 70101, Taiwan; 5 Department of Cell Biology and Anatomy, College of Medicine, National Cheng Kung University, Tainan 70101, Taiwan

**Keywords:** astrocyte, obesity, depression, GLAST, GLT-1

## Abstract

Background/Objectives: Depression is frequently comorbid with obesity. We previously showed that astrocyte-mediated hyperactive ventral hippocampal glutamatergic afferents to the nucleus accumbens determined the exhibition of depression-like behaviors in obese murine models. However, it remains unclear if the metabolic disorder-induced depressive phenotypes and astrocytic maladaptation in the ventral hippocampus (vHPC) could be reversed following the amelioration of key metabolic impairments such as insulin resistance and dyslipidemia. Method: Male mice were fed a high-fat diet (HFD) for 12 weeks, followed by either continued HFD feeding (HFD/HFD group) or a switch to a standard diet for 4 weeks (HFD/SD group). Results: Results showed that HFD/HFD mice displayed not only glucose/lipid metabolic dysfunction, but also depression-like behaviors. In contrast, HFD/SD mice showed improvements in metabolic disorders and depressive phenotypes. Mechanistically, dietary fat reduction restored astrocyte morphology and glutamate transporter expression (GLT-1, GLAST) in the vHPC and suppressed neuroinflammatory signaling, as evidenced by reduced levels of phospho-IKK, TNF-α, IL-1β, and IL-6 in the vHPC. Conclusions: These findings suggest that dietary fat reduction reverses obesity-induced depressive phenotypes, astrocytic deficits, at least in part via suppression of neuroinflammation through the NF-κB signaling pathway.

## 1. Introduction

Obesity, defined by an abnormal or excessive body fat (BF) accumulation, has become an increasing public health problem worldwide over the past two decades, with approximately 50% of U.S. adults expected to meet clinical criteria for obesity by 2030 [1,2]. Major depressive disorder (MDD), clinically defined by the presence of continuous low mood or anhedonia lasting for a minimum of two weeks, is commonly observed in people with obesity, as well as in animal models genetically predisposed to excessive weight gain [3,4]. Obesity and depression are two highly prevalent and often comorbid conditions that pose significant public health challenges worldwide [5]. Numerous epidemiological studies have demonstrated a strong bidirectional association between obesity and depression, wherein individuals with obesity have a higher risk of developing depression, and vice versa [5,^6^]. Epidemiological data suggest that approximately 43% of adults diagnosed with MDD are obese, and obesity is significantly higher among individuals with psychiatric disorders compared to those without mental health conditions [4]. Animal studies also support such an association. Mice with obesity and/or diabetes frequently display behavioral phenotypes indicative of depressive-like and anxiety-like states [7,8,9].

The hippocampus, particularly its ventral region, plays a central role in the regulation of emotion and affective behaviors, and has been strongly implicated in the pathophysiology of depression. Astrocytes within the hippocampus are increasingly recognized as key modulators of synaptic transmission, metabolic support, and neuroinflammatory responses—processes that are frequently disrupted in depressive states [10,11]. Notably, postmortem studies and preclinical models of depression have demonstrated significant reductions in hippocampal astrocyte density and function, suggesting a region-specific glial contribution to mood regulation [12]. Therefore, we focused our investigation on hippocampal astrocytes to elucidate their potential involvement in the astroglial mechanisms underlying obesity-associated depressive-like behaviors.

Obesity has been shown to induce chronic low-grade systemic inflammation [13], such as elevated concentrations of circulating proinflammatory cytokines and enhanced infiltration of macrophages within white adipose tissue [14]. Obesity is also associated with central inflammation. It has been shown that levels of TNF, IL-1β, and IL-6, as well as activation of the NF-κB signaling pathway within the hypothalamus, are elevated in obese rats [15]. Peripheral and central inflammations are also evident in patients with depression and depression animal models [16,17]. Compared to non-depressed controls, patients with MDD have increased expression of IL-6 in the blood and activation of NF-kB in the peripheral mononuclear cells [16]. In rodent models of depression induced by chronic unpredictable stress, expressions of TNF, IL-1β, IL-6, cyclooxygenase-2 (COX-2), and inducible nitric oxide synthase (iNOS) are elevated in the hippocampus [17]. Interestingly, trifluoperazine, an antipsychotic drug, inhibits neuroinflammation in the hypothalamus of obese mice [18,19]. These results indicate an intriguing role of inflammation in the interaction between obesity and depression.

Previously, we have demonstrated that astrocytic malfunction determines the manifestation of depression-like behavior in a DIO mouse model [9]. In that study, mice were fed with an HFD for 12 weeks to induce obesity and depressive phenotypes, and it was found that hyperactivity of ventral hippocampal glutamatergic projections to the nucleus accumbens was found to be a key driver of depression-like behavior in these mice. Furthermore, our findings demonstrated that reduced expression of two glutamate transporters, GLAST and GLT-1, resulted in circuit-level maladaptations. GLAST and GLT-1, predominantly localized in astrocytes, are responsible for the vast majority of synaptic glutamate clearance in the central nervous system [20]. Studies have indicated that individuals with MDD have diminished expression of glutamate transporters, EAAT1 and EAAT2 (alternative terms of GLAST and GLT-1 in humans) [21]. DIO mice also displayed reduced levels of GLT-1 and GLAST in the hippocampi [7,8,9]. Prolonged consumption of HFD causes astrocyte shrinkage, which afterward damages their ability to regulate neurotransmission [22]. In the brains of individuals with severe depression, the number of astrocytes is decreased [11]. Atrophy of hippocampal astrocytes is also observed in corticosterone-induced depressive mice [23].

Diet-induced weight loss is one of the most widely recommended measures for obese persons. Diet-induced weight loss is known to reduce the risk of metabolic syndromes, improve mental health, and ameliorate overall inflammation in obese individuals [19]. However, whether diet-induced weight loss can reverse HFD-induced astrocytic maladaptation and associated behavioral deficits is untested. To address this issue, we first fed 8-week-old mice with an HFD for 12 weeks to induce metabolic disorders and depression-like behaviors as previously described [8,9]. Then the HFD was switched to a standard diet (SD) for 4 weeks to induce weight loss (termed HFD/SD group). Mice without switching HFD (termed HFD/HFD group) served as the DIO controls, while mice fed with an SD for 16 weeks served as normal controls (SD/SD mice). Their metabolic endpoints (e.g., body weight, wet white and brown adipose weight, liver weight, fasting blood glucose and plasma insulin, and glucose tolerance test), depressive phenotypes, astrocytic morphology, and inflammation in the vHPC were determined and compared among the three groups.

## 2. Materials and Methods

### 2.1. Animals

Male C57BL/6N mice were purchased from the National Cheng Kung University Laboratory Animal Center. Mice were housed in ventilated cages (4–5 per cage) in a temperature-controlled (24 ± 1 °C) and humidity-controlled (55 ± 10%) specific-pathogen-free breeding unit of NCKULAC on a 12-h light/12-h dark cycle (light on at 7 A.M.). Mouse studies were approved by the Institutional Animal Care and Use Committee (IACUC) of National Cheng Kung University (Approval No. 109148) and were conducted in accordance with both institutional and national ethical guidelines.

#### Feeding Regimens

**Figure 1:**
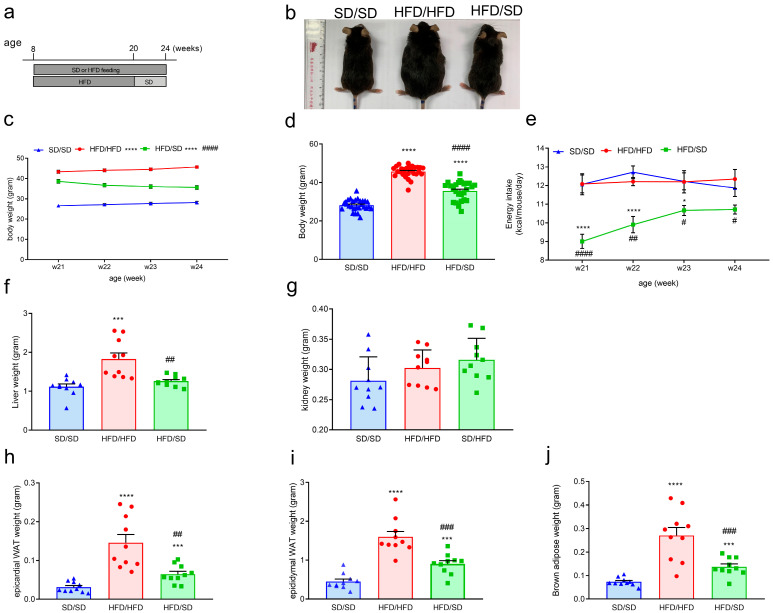
(**a**–**j**) Effects of the reduction of dietary fat on body weight and organ weight. (**a**) Experimental timeline. (**b**) Appearance. (**c**) Quantitative results of body weight of mice during the feeding period. (**d**) Quantitative results of body weight of mice after the end of the regimen. (**e**) Quantitative results of energy intake of mice. (**f**) Liver weight, (**g**) kidney weight. (**h**) Epicardial white adipose tissue. (**i**) Epididymal white adipose tissue. (**j**) Brown adipose tissue. * *p* < 0.05, , *** *p* < 0.001, **** *p* < 0.0001, vs. respective SD/SD group. # *p* < 0.05, ## *p* < 0.01, ### *p* < 0.001, #### *p* < 0.0001, vs. respective HFD/HFD group. Numbers given in parentheses indicate the sample sizes.

Male C57BL/6N mice at the age of 8 weeks were randomly assigned to a standard diet (SD) group or an HFD group. The SD mice received a standard chow diet (Cat. #: 5010, LabDiet, St. Louis, MO, USA) for 16 weeks (termed SD/SD group), and the HFD mice were provided with a diet comprising 60% of total caloric intake from fat (Cat. #: 58Y1, TestDiet, St. Louis, MO, USA) for 12 weeks. Following 12 weeks of HFD feeding, the HFD-fed mice were randomly and equally divided into two groups: (1) continued to feed with HFD (termed HFD/HFD group) and (2) switched to SD (termed HFD/SD group) for four more weeks. The total feeding period was 16 weeks. Food intake and body weight were evaluated weekly throughout the experimental period. The experimental timelines are shown in Figure 1a. The number of mice used in each experiment is listed in Supplementary Figure S1.

### 2.2. Intraperitoneal Glucose Tolerance Test

Following 16 weeks of HFD treatment, an intraperitoneal glucose tolerance test (IPGTT) was administered in accordance with modified protocols from previous studies [8,9]. After fasting for 12 h, each animal was administered an intraperitoneal injection of a sterile 40% glucose solution, prepared in saline, at a dosage of 2 g/kg body weight. Peripheral blood samples were collected via tail vein puncture at baseline (0 min) and at 15, 30, 60, 90, and 120 min post-injection. Blood glucose level was determined using a handheld glucometer (OneTouch^®^ UltraEasy (LifeScan, Milpitas, CA, USA)) in conjunction with compatible test strips.

### 2.3. Intraperitoneal Insulin Tolerance Test

An intraperitoneal insulin tolerance test (IPITT) was conducted after 16 weeks of dietary HFD, with reference to established methodologies [8,9]. After fasting for 4 h, each animal received an intraperitoneal injection of human insulin at a dosage of 0.75 U/kg body weight. Capillary blood samples were collected from the tail vein at designated time points: 0, 15, 30, 60, 90, and 120 min post-injection. Blood glucose concentrations were assessed using a glucometer and corresponding OneTouch^®^ Ultra test strips.

### 2.4. Measuring Fasting Plasma Levels of Glucose and Insulin

Blood was obtained from mice via cardiac puncture using capillary tubes coated with heparin after 12 h fasting. The plasma fraction was separated by centrifugation at 3000× *g* for 10 min at 4 °C. Plasma glucose concentrations were quantified using a commercially available glucose-oxidase kit. Plasma insulin levels were examined using an insulin ELISA kit.

### 2.5. Sucrose Preference Test

The sucrose preference test (SPT) was conducted in accordance with previously established protocols [8,9]. To eliminate potential confounding variables associated with individual housing or the reassignment of cage mates, the SPT was performed on a per-cage basis. Mice were offered access to two identical water bottles for a total duration of 24 h. One contained distilled water, and the other contained a 1% (*w*/*v*) sucrose solution prepared in distilled water. To minimize side preference, the positions of the two bottles were exchanged after 12 h. Fluid intake was quantified by weighing the bottles before and after the experimental period. Sucrose preference was calculated as the proportion of sucrose solution intake relative to the total fluid intake (sucrose solution plus water) over the 24 h period.

### 2.6. Forced Swimming Test

The forced swimming test (FST) was carried out in accordance with previously established protocols [8,9]. Each mouse was individually placed in a transparent cylindrical tank measuring 30 cm in height and 20 cm in diameter, containing 15 cm of water maintained at a temperature between 23 °C and 25 °C. Behavioral observations were conducted over a 6 min testing period, during which active behaviors (such as swimming and climbing) and passive responses (immobility) were assessed. Only data collected from the final 4 min were included in the subsequent analysis.

### 2.7. Tail Suspension Test

The tail suspension test (TST) was carried out in accordance with previously reported procedures [8,9]. Mice were suspended by the tail at a height of 50 cm above the base of a white plastic enclosure, with the animal positioned in an inverted posture for a total duration of 6 min. Behavioral activity was continuously recorded throughout the test, but only data from the final 4 min were included in the subsequent analysis to minimize handling-induced artifacts.

### 2.8. Measurement of Plasma Lipid Concentrations

Plasma lipid levels were examined using enzyme-linked immunosorbent assay (ELISA) kits. The assays included quantification of free fatty acids (Cat. #: K612-100), triglycerides (Cat. #: K622-100), high-density lipoprotein (HDL) cholesterol (Cat. #: K613-100), and low-density lipoprotein (LDL) cholesterol (Cat. #: K613-100).

### 2.9. Tissue Preparations

The mice were anesthetized and transcardially perfused via the left ventricle using ice-cold saline. Following perfusion, brains were extracted and separated into left and right hemispheres. For immunoblotting analysis, the ventral hippocampus (vHPC) was rapidly isolated, snap-frozen in liquid nitrogen, and preserved at −80 °C until needed. For RNA extraction, the vHPC specimens were soaked in TRIzol reagent and subsequently subjected to total mRNA extraction. For immunohistochemical analysis, the brain hemispheres were fixed in 4% paraformaldehyde prepared in 0.1 M phosphate buffer at 4 °C for 48 h. The fixed tissue was then passed through graded sucrose solutions (10%, 20%, 30%, and 35% prepared in 0.1 M phosphate buffer) for dehydration, followed by embedding in optimal cutting temperature (OCT) compound. Finally, brains were coronally sectioned at 25 μm thickness and preserved in cryoprotectant at −20 °C until further use.

### 2.10. Immunoblotting

The vHPC tissues were homogenized in a chilled commercial lysis buffer containing a protease inhibitor cocktail tablet. The total protein concentration was measured using the Bradford method (Bio–Rad protein assay). Equal amounts of extracted protein samples were loaded onto SDS-PAGE gels for electrophoretic separation and subsequently transferred onto the polyvinylidene difluoride (PVDF) membranes. Membranes were blocked in blocking buffer, then incubated overnight at 4 °C with primary antibodies, including anti-GFAP (Cat. #: Z0334), anti-GLAST (Cat. #: D44E2), anti-GLT-1 (Cat. #: 3838), anti-pIKKα/β (Cat. #: 2697), anti-IKKβ (Cat. #: 8943), anti-GAPDH (Cat. #: 30396), and anti-actin (Cat. #: A5441). Following washes, membranes were exposed to horseradish peroxidase-conjugated secondary antibodies. Detection of protein bands was carried out using enhanced chemiluminescence (ECL) reagents and signal intensity was quantified using the ImageQuant LAS 4000 imaging system.

### 2.11. Real-Time Quantitative PCR Analysis

Total RNAs were extracted using TRIzol reagent. The reverse transcription reaction was carried out using 2 μg of RNA with SuperScript III First-stand Synthesis System and random hexamer primers. Quantitative PCR was performed using SYBR Green dye to detect amplification products. Gene expression levels were quantified using a real-time PCR system. Target mRNA levels were normalized to β-actin, which served as the endogenous control. Primer sequences are provided in Supplementary Table S1.

### 2.12. Immunohistochemistry

Brain sections containing the ventral hippocampus (vHPC) were obtained based on stereotaxic coordinates ranging from 2.30 mm to 3.88 mm posterior to the Bregma. To eliminate residual embedding compounds from the frozen tissue, the collected slices were washed in phosphate-buffered saline (PBS) supplemented with 0.3% Triton X-100. To quench endogenous peroxidase activity, sections were treated with 3% hydrogen peroxide (H_2_O_2_) solution. Non-specific antibody binding was minimized by pre-incubation with 3% normal goat serum for 1 h at room temperature. Subsequently, the free-floating brain sections were incubated overnight at 4 °C with anti-GFAP antibody. After thorough washing, sections were incubated with secondary antibodies for 2 h at room temperature. The brain sections were washed and applied for a 3,3′-diaminobenzidine kit.

### 2.13. Analysis of Astrocytic Morphology

Photomicrographs were acquired using a digital camera connected to a computer running Axiovision 4.8 software (Carl Zeiss, Oberkochen, Germany). To reduce batch-to-batch variation, all immunohistochemical procedures were performed simultaneously under identical conditions. The GFAP-immunoreactive (GFAP^+^) signals were detected by identifying pixels with intensities exceeding a predetermined background threshold using the Fiji distribution of ImageJ (v2.0.0-rc-69/1.52p, U.S. National Institutes of Health). A uniform background intensity threshold was established and consistently applied across all tissue sections. The ImageJ plugin, NeuronJ, was employed to quantify the process arbors (Sholl analysis), process lengths, and branch points of the GFAP-positive astrocytes in the CA1 region of the ventral hippocampus. For each mouse, a total of 25 astrocytes (five astrocytes per brain section from five serial brain sections) were analyzed. The averaged value of measured parameters obtained from the 25 selected astrocytes was presented as one data point. A total of five animals per experimental group were evaluated. All morphological assessments were performed by an experimenter who was blinded to the group assignments to minimize observational bias.

### 2.14. Statistical Analysis

All statistical analyses were conducted using GraphPad Prism 8.0 (GraphPad Software, Inc., San Diego, CA, USA). The number of biological replicates (N) applied in each experimental condition is detailed in the respective figure legends. Results are expressed as the mean ± standard deviation (SD). A *p*-value less than 0.05 was considered statistically significant. Student’s *t*-test was employed to analyze the data sets with a single factor. One-way ANOVA followed by Tukey’s multiple comparisons was used to analyze differences between more than two independent groups. The body weight and energy intake of mice were analyzed using repeated-measure two-way ANOVA. The process of arborization of individual astrocytes (Sholl analysis) was analyzed using mixed-model two-way ANOVA with concentric rings and HFD as the two main effects. Sidak’s post-hoc test was used to perform multiple comparison analysis after the two-way ANOVAs.

## 3. Results

### 3.1. A 12-Week High-Fat Diet (HFD) Feeding Induces Obesity, Metabolic Dysfunction, and Depression-like Behaviors in Mice

To establish the DIO-related depression mouse model, 8-week-old male C57BL/6N mice were administered with an HFD over a period of 12 weeks to induce obesity and a series of metabolic dysregulations (Supplementary Figure S2a). Compared to animals maintained on standard chow (SD), HFD-fed mice displayed a progressive and significant increase in weight gain throughout the feeding period (Supplementary Figure S2b), culminating in an approximately 50% higher body weight at the end of 12-week feeding (Supplementary Figure S2c). No statistically significant differences in energy intake were observed between mice maintained on an HFD and those receiving a SD (Supplementary Figure S2d). To evaluate metabolic status, IPGTT and IPITT were conducted. Mice subjected to the HFD regimen displayed impaired glucose clearance (Supplementary Figure S2e) and reduced insulin responsiveness (Supplementary Figure S2f). Furthermore, elevated fasting plasma glucose (Supplementary Figure S2g), increased circulating insulin levels (Supplementary Figure S2h), and a higher HOMA-IR index (Supplementary Figure S2i) were observed in the HFD mice, indicating the presence of systemic insulin resistance.

Given the well-documented link between metabolic disturbances and mood disorders [24,25,26], we next examined behavioral changes associated with depressive-like behaviors using the SPT, FST, and TST. HFD-fed mice showed a significant reduction in sucrose preference (Supplementary Figure S3a), as well as increased immobility durations in both the FST (Supplementary Figure S3b) and TST (Supplementary Figure S3c). These data showed that a 12-week HFD feeding results in obesity, systemic insulin resistance, and depression-like behaviors in mice.

### 3.2. Reduction of Dietary Fat Ameliorates HFD-Induced Obesity, Systemic Insulin Resistance, and Hyperlipidemia in Mice

**Figure 2:**
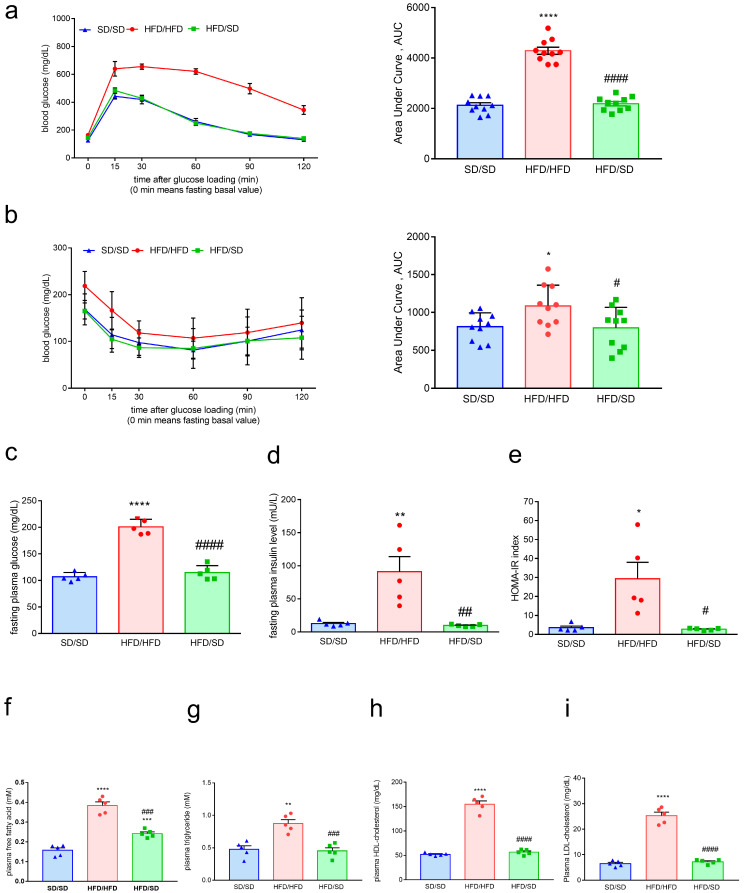
Effects of the reduction of dietary fat on systemic glucose and lipid metabolism. (**a**) Alterations of blood glucose levels of mice in the IPGTT shown in the left panel, and quantitative results of AUC shown in the right panel. (**b**) Alterations of blood glucose levels of mice in the IPITT shown in the left panel, and quantitative results of AUC shown in the right panel. (**c**) Quantitative results of fasting plasma levels of glucose in mice. (**d**) Quantitative results of fasting plasma levels of insulin in mice. (**e**) Quantitative results of the HOMA-IR index. (**f**) Quantitative results of plasma levels of free fatty acid in mice. (**g**) Quantitative results of plasma levels of triglyceride in mice. (**h**) Quantitative results of plasma levels of HDL cholesterol in mice. (**i**) Quantitative results of plasma levels of LDL/VLDL cholesterol in mice. * *p* < 0.05, ** *p* < 0.01, **** *p* < 0.0001, vs. respective SD/SD group. # *p* < 0.05, ## *p* < 0.01, ### *p* < 0.001, #### *p* < 0.0001, vs. respective HFD/HFD group. Numbers given in parentheses indicate the sample sizes.

Next, we examined whether the reduction of dietary fat improved HFD-induced glucose metabolic disturbances. After a 12-week HFD regimen, mice were transitioned to a standard chow diet (SD) for an additional 4 weeks (Figure 1a). Our data showed that the body weight of HFD/SD mice was significantly decreased when compared with that of HFD/HFD mice (Figure 1b–d). The weights of organs, including the liver, epicardial white adipose tissue (WAT), epididymal WAT, and brown adipose tissue (BAT), were markedly decreased in HFD/SD mice compared with HFD/HFD mice (Figure 1f,h–j). In contrast, kidney weight did not differ significantly between the groups (Figure 1g). To evaluate the effect of switching diet from an HFD to an SD on the systemic glucose handling, an IPGTT was conducted. HFD/SD mice exhibited more efficient glucose clearance compared to HFD/HFD mice, indicating change of the dietary fat improved glucose tolerance (Figure 2a). Correspondingly, results from the IPITT demonstrated enhanced insulin sensitivity in the HFD/SD mice compared to HFD/HFD mice (Figure 2b). Furthermore, fasting plasma glucose levels and fasting plasma insulin levels, along with HOMA-IR index, were significantly reduced in the HFD/SD mice compared to their HFD/HFD counterparts (Figure 2c–e). Biochemical analysis of plasma further revealed elevated levels of free fatty acids, triglycerides, and total cholesterol in the HFD/HFD group (Figure 2f–i). Reduction of dietary fat reversed these metabolic dysregulations (Figure 2f–i). Our results showed that the reduction of dietary fat ameliorates HFD-induced metabolic dysfunctions in mice.

### 3.3. Reduction of Dietary Fat Improves HFD-Induced Depressive Phenotypes in Mice

**Figure 3:**
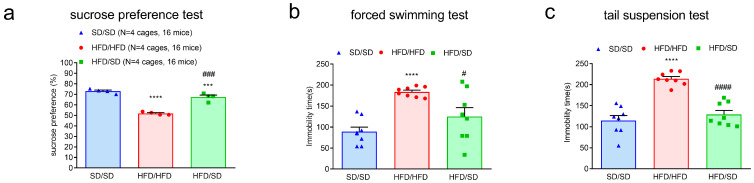
Effects of the reduction of dietary fat on the exhibition of depression-like behaviors in mice. (**a**) Quantitative results of SPT. (**b**) Quantitative results of FST. (**c**) Quantitative results of TST. *** *p* < 0.001, **** *p* < 0.0001, vs. respective SD/SD group. # *p* < 0.05, ### *p* < 0.001, #### *p* < 0.0001, vs. respective HFD/HFD group. Numbers given in parentheses indicate the sample sizes.

To investigate the potential impact of dietary fat reduction on depressive-like behaviors, behavioral assessments were performed in mice subjected to different dietary regimens. Mice maintained on an HFD exhibited behavioral alterations consistent with depressive-like phenotypes, as evidenced by reduced sucrose preference in the SPT (Figure 3a) as well as increased immobility durations in both the FST and the TST (Figure 3b,c). A 4-week switching diet from an HFD to an SD resulted in a significant improvement in affective behavior. Specifically, the dietary reversal restored sucrose consumption levels in the SPT (Figure 3a) and significantly decreased immobility times in the FST and TST (Figure 3b,c). These findings suggest that the reduction of dietary fat intake effectively attenuates HFD-induced depressive-like behaviors in mice.

### 3.4. Reduction of Dietary Fat Attenuates the HFD-Induced Astrocyte Activation and Astrocytic Morphology Remodeling in the Hippocampi of Mice

**Figure 4:**
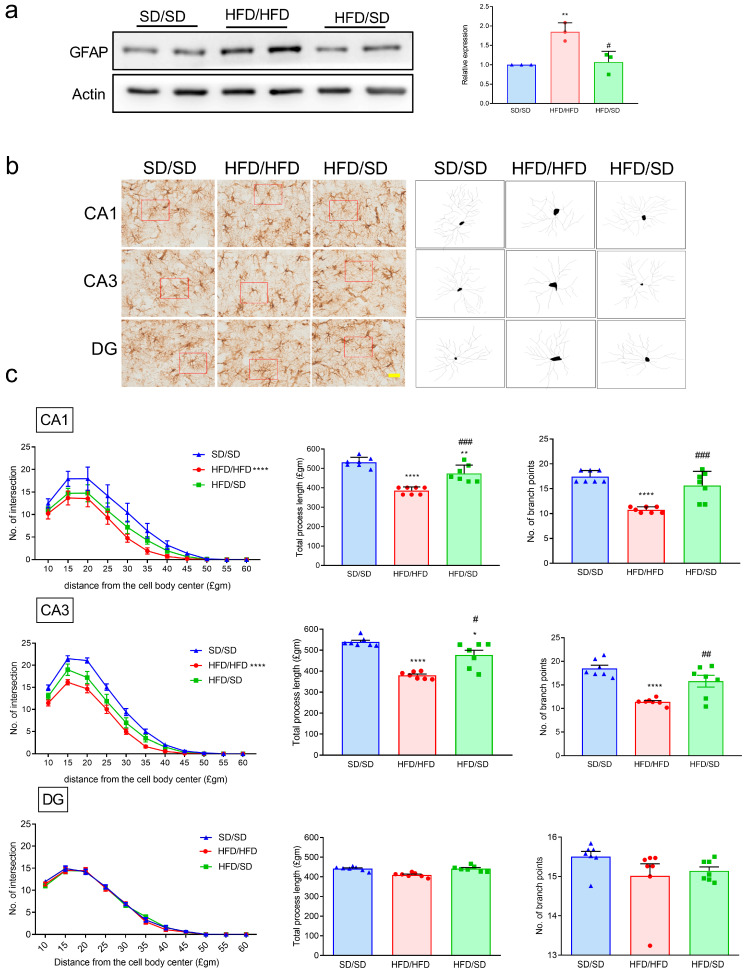
Effects of the reduction of dietary fat on GFAP expression and astrocytic fine structures in the hippocampus of mice. (**a**) Representative immunoblotting micrographs of GFAP in the hippocampus of mice (left panels). Quantitative results of GFAP levels in the hippocampus of mice (right panels). (**b**) Representative immunomicrographs of the GFAP^+^ astrocytes in the hippocampus (left nine panels). Representative process tracing graphs of selected GFAP^+^ astrocyte (red frames in the left nine panels) are shown in the respective nine right panels. (**c**) Quantitative results of the Sholl analysis, total process lengths, and numbers of branch points. * *p* < 0.05, ** *p* < 0.01, **** *p* < 0.0001, vs. respective SD/SD group. # *p* < 0.05, ## *p* < 0.01, ### *p* < 0.001, vs. respective HFD/HFD group, ordinary two-way ANOVA in scatter plots and mixed-model repeated measure two-way ANOVA in line graphs. Numbers given in parentheses indicate the sample sizes. Values represent mean ± S.E.M. from three independent experiments.

Our previous studies showed that HFD not only induces depressive-like behaviors but also triggers astrocyte activation and morphological remodeling within the hippocampus [8,9]. In the present study, we assessed whether the reduction of dietary fat, specifically transitioning from an HFD to an SD, could reverse HFD-induced astroglial alterations. The ventral hippocampus (vHPC) was chosen in this study due to its well-established role in mood regulation and its involvement in the pathophysiology of affective disorders such as anxiety and depression. In line with our previous findings [8,9], mice maintained on an HFD exhibited elevated expression of glial fibrillary acidic protein (GFAP) in the ventral hippocampus, indicative of astrocyte activation (Figure 4a). Notably, this upregulation was abolished in mice that underwent dietary fat reduction through SD intervention, suggesting that astrocytic activation was mitigated by the nutritional shift (Figure 4a). Given that astrocyte activation is frequently accompanied by morphological adaptations, such as hypertrophy and structural remodeling, we further analyzed the effects of switching from an HFD to an SD diet on the astrocytic architecture using Sholl analysis. Results revealed a significant reduction in total process lengths, the number of branch points, and the number of process intersections with concentric rings in the Sholl analysis of the GFAP^+^ astrocytes located in the ventral hippocampal CA1 and CA3 of HFD/HFD mice (Figure 4b,c). HFD-induced process remodeling of the ventral hippocampal CA1 and CA3 astrocytes was reversed in HFD/SD mice (Figure 4b,c). There was no difference in the DG region between each group (Figure 4b,c). Our results suggested that switching diet from an HFD to an SD increases astrocytic process arborization in mice.

### 3.5. Reduction of Dietary Fat Reverses the HFD-Downregulated Astrocytic Neuroplasticity-Related Proteins in the Hippocampi of Mice

**Figure 5:**
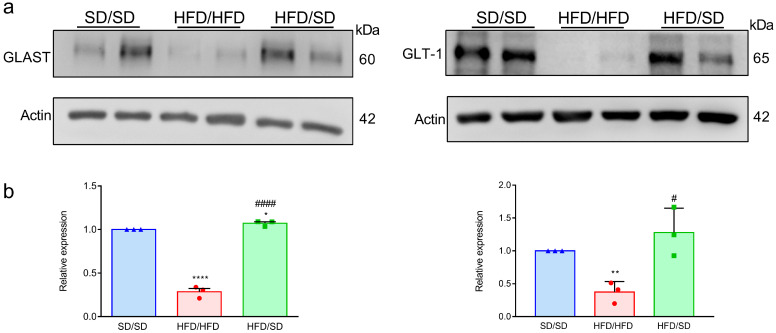
Effects of the reduction of dietary fat on expression levels of neuroplasticity-related proteins in the hippocampus. (**a**) Representative immunoblotting micrographs. (**b**) Quantitative results. * *p* < 0.05, ** *p* < 0.01, **** *p* < 0.0001, vs. respective SD/SD group. # *p* < 0.05, #### *p* < 0.0001, vs. respective HFD/HFD group, unpaired Student’s *t* test. n = 6 mice, values represent mean ± S.E.M. from three independent experiments.

Neuronal plasticity-related protein, GLT-1 and GLAST are two glutamate transporters, which take part in the reuptake of glutamate around the synaptic cleft to prevent excitotoxicity. Previous studies reported that the levels of hippocampal GLT-1 and GLAST were both significantly diminished in MDD patients compared to healthy subjects [21]. Recent work by Tsai et al. reported that 12 weeks of HFD feeding diminished the levels of GLAST and GLT-1 in the hippocampus of mice [9]. The effect of switching diet from an HFD to an SD on the expression of astrocyte-associated proteins involved in neuroplasticity within the hippocampal region was investigated. The level of GLAST and GLT-1 was decreased in the hippocampus of HFD/HFD mice compared to that of the SD/SD group (Figure 5). The HFD-downregulated GLAST and GLT-1 expressions were reversed in the hippocampi of HFD/SD mice (Figure 5).

### 3.6. Reduction of Dietary Fat Abolishes the HFD-Induced Inflammatory Factors in the Ventral Hippocampus of Mice

**Figure 6:**
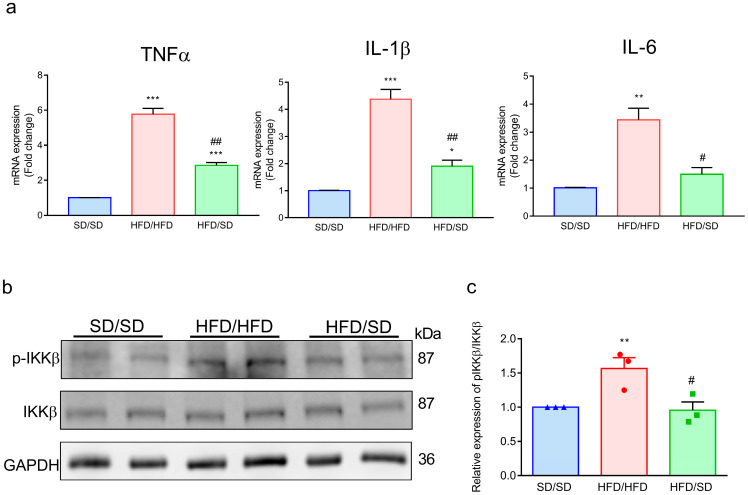
Effects of the reduction of dietary fat on inflammatory factors in the hippocampus. (**a**) Expression of TNF-α, IL-1, and IL-6 was examined by real-time quantitative PCR. Each value represents mean ± SEM from at least three mice in each group. * *p* < 0.05, ** *p* < 0.01, *** *p* < 0.001, vs. respective SD/SD group. # *p* < 0.05, ## *p* < 0.01 vs. respective HFD/HFD group. (**b**) Representative immunoblotting micrographs. (**c**) Quantitative results. ** *p* < 0.01, # *p* < 0.05, unpaired Student’s *t* test. n = 6 mice, values represent mean ± S.E.M. from three independent experiments.

Accumulating evidence supports a strong link between neuroinflammatory processes and the pathophysiology of depressive disorders [26]. Emerging studies further highlight a close association between the development and progression of depression and the involvement of key proinflammatory cytokines IL-1β, TNF-α, and IL-6 [27,28,29]. Astrocytic IKKβ/NF-κB was upregulated in the ventromedial hypothalamus by chronic overnutrition, resulting in impaired astrocytic plasticity and sustained shortening of astrocytic processes [30]. Our results showed that hippocampal expression levels of the proinflammatory cytokines TNF-α, IL-1β, and IL-6 were markedly elevated in HFD/HFD mice relative to SD/SD mice (Figure 6a & Supplementary Figure S5). Notably, transitioning from an HFD to a standard diet (HFD/SD) led to a significant attenuation of these cytokine levels, indicating a reduction in neuroinflammatory signaling within the hippocampus. We further examined whether IKKβ/NF-κB might contribute to the control of hippocampal astrocytic process changes. We observed increased phospho-IKKβ in the ventral hippocampus of HFD/HFD mice compared with that of SD/SD mice (Figure 6b,c). The HFD-induced high level of phospho-IKKβ was abolished in the ventral hippocampus of HFD/SD mice (Figure 6b,c).

## 4. Discussion

This study highlights the potential therapeutic benefits of the reduction of dietary fat on both T2DM and its comorbid depressive-like behaviors. Our data suggested that reduction of dietary fat mitigates the HFD-induced depressive-like phenotypes and astrocytic impairments in the ventral hippocampus may be mediated through suppression of the IKKβ/NF-κB signaling cascade. This conclusion is substantiated by the following lines of evidence: (1) Reduction of dietary fat ameliorated HFD-induced metabolic disturbances; (2) Reduction of dietary fat enhanced sucrose preference in the SPT and decreased immobility in the FST and TST, respectively; (3) Reduction of dietary fat significantly reduced hippocampal expression of GFAP, a marker of astrocyte activation; (4) Reduction of dietary fat restored astrocytic structural complexity, including process arborization of GFAP-positive cells in the CA1 and CA3 regions of the ventral hippocampus; (5) Reduction of dietary fat reversed the HFD-downregulated astrocytic neuroplasticity-associated proteins in the hippocampi of mice; (6) Reduction of dietary fat abolished the HFD-induced phospho-IKKβ in the ventral hippocampus of mice.

A growing body of evidence links metabolic dysfunctions to the development of MDD [5,6]. Nonetheless, the precise mechanisms underlying the interactions between insulin resistance and neuropsychiatric disorders remain incompletely understood. Previous studies have confirmed that weight loss by caloric restriction can enhance systemic energy homeostasis and improve adipose tissue functionality [31]. In this study, we observed that prolonged exposure to an HFD led to the emergence of both metabolic abnormalities and depression-like behaviors in obese mice, aligning with previously reported observations [8,9]. Our data showed that 12 weeks of HFD consumption resulted in significant metabolic disturbances, including increased body weight, impaired insulin sensitivity, and disrupted lipid metabolism. Biochemical analyses revealed elevated circulating levels of glucose, insulin, total cholesterol, and triglycerides in HFD/HFD mice. These metabolic dysregulations were improved in the HFD/SD mice. In addition to metabolic improvements, the reduction of dietary fat also ameliorated HFD-induced behavioral changes associated with depression. It suggested that reduction of dietary fat could improve HFD-induced depression-like behavior, potentially through mechanisms involving improved insulin signaling and normalization of lipid homeostasis. The present findings have important clinical implications. Given the increasing prevalence of comorbid obesity and depression, our results suggest that dietary interventions targeting fat intake may represent a non-pharmacological strategy to mitigate depressive symptoms associated with metabolic dysregulation. The reversal of astrocytic morphological impairments and behavioral deficits following dietary fat reduction highlights the potential for lifestyle-based approaches to restore brain glial function and emotional regulation. Unlike pharmacotherapy, which may be limited by side effects or non-responsiveness in some individuals, dietary modulation offers a feasible, cost-effective, and accessible intervention. These findings underscore the therapeutic relevance of dietary composition—not merely caloric intake—in addressing the neurobiological underpinnings of obesity-related mood disorders, and warrant further exploration in clinical settings.

Disruption of the capacity for neuroplasticity could contribute to a range of hippocampal-dependent behavioral disturbances, including depressive symptoms, anxiety, and cognitive decline. Astrocytes act as support cells for neuroplastic processes. In agreement with the present findings, previous studies have reported that HFD exposure elicits astrocyte activation. For instance, prolonged HFD intake (16 weeks) has been shown to increase GFAP expression and expand the area occupied by GFAP-positive astrocytes in the cerebellum, while no such changes were detected in the cerebral cortex [32]. Interestingly, astrocytic morphology appears to be differentially affected across brain regions: in the CA1 subfield of the hippocampus, long-term HFD feeding (12 months) led to a reduction in the number of astrocytic processes [33], whereas an increase in total process length was observed in the CA3 region following 11 months of HFD exposure [34]. These findings highlight the region-specific sensitivity of astrocytes to dietary fat-induced alterations. Herein, we demonstrated that the reduction of dietary fat could reverse the altered astrocytic morphological remodeling as evidenced by an increase in the total process length and a greater number of branch points. We found no significant differences in astrocyte density across SD/SD, HFD/HFD, and HFD/SD groups (Supplementary Figure S5). These results suggest that HFD does not alter the number of GFAP⁺ astrocytes per se, but rather induces structural remodeling that may compromise astrocytic function. Importantly, dietary fat reduction effectively reversed these morphological abnormalities, as indicated by restored process complexity and increased branching in the HFD/SD group. Furthermore, we observed that chronic HFD consumption reduced the expression of key astrocytic glutamate transporters, GLAST and GLT-1, in the hippocampus of mice. Such reductions may compromise glutamate clearance, potentially leading to hyperactivity within glutamatergic projections from the hippocampus to the nucleus accumbens. Reduction of dietary fat could reverse HFD-induced downregulated GLAST and GLT-1 expression. These results imply that the glial glutamate transporters in the hippocampus may constitute a critical component in the pathogenesis of depression. In support of this premise, patients with major depressive disorder displayed lower hippocampal expression of EAAT1 and EAAT2, the human homologs of GLAST and GLT-1, than age-matched control individuals [21]. Taken together, our findings suggest that dietary fat reduction mitigates HFD-induced depressive-like behaviors by restoring astrocyte-related glutamatergic homeostasis in the ventral hippocampus.

Neuroinflammation has been shown to be highly associated with the etiology of depression [27]. Chronic stress could stimulate the release of proinflammatory cytokines, including TNF-α, IL-1β, and IL-6. Studies have demonstrated markedly elevated concentrations of proinflammatory cytokines in the hippocampal tissue of individuals with depression who died by suicide, compared to non-depressed controls [35]. Consistent with these findings, our results demonstrate significantly increased levels of TNF-α, IL-1β, and IL-6 in the hippocampus of HFD/HFD mice. Importantly, dietary reversal (HFD/SD) led to a substantial attenuation of these cytokines, suggesting that reducing dietary fat intake may suppress hippocampal neuroinflammatory signaling. NF-κB is a broadly expressed transcription factor that regulates the expression of a wide variety of genes implicated in diverse cellular activities [36]. Dysregulation of NF-κB signaling has been associated with impairments in neurogenesis, neuronal structure, cognitive functions, and affective behaviors [37]. A recent study done by Zhang et al. demonstrated that a reduction in the length of astrocytic processes within the hypothalamus in HFD-fed mice was associated with moderate-level IKKβ/NF-κB upregulation [17]. Zhang et al. showed that activation of IKKβ/NF-κB signaling cascade led to a marked retraction of astrocytic processes, while inhibition of IKK/NF-κB reversed this process defect in obese mice by using genetic models, thereby implicating the IKK/NF-κB signaling cascade plays an important role in the regulation of astrocytic processes [17]. In alignment with our findings, heightened NF-κB activity was observed in the hippocampus of HFD/HFD mice. However, reduction of dietary fat led to inhibitory effects on NF-κB activation in the hippocampus, which was illustrated by reduced phospho-IKKβ, suggesting that modifying dietary fat may confer neuroprotective benefits by mitigating hippocampal inflammation associated with obesity-induced chronic low-grade inflammatory states. However, it remains unclear whether astrocytes are the primary cellular source of the observed inflammatory cytokines and NF-κB activation. Although astrocytes are key regulators of neuroinflammatory responses and may plausibly contribute to the elevated cytokine levels and NF-κB signaling, proinflammatory cytokines are secreted proteins, and bulk tissue-level measurements do not allow for definitive identification of their cellular origin. The concurrent astrocytic morphological changes observed in our study suggest possible involvement of astrocytes in mediating these inflammatory responses, but additional immunofluorescence or cell-type-specific analyses are needed to confirm this hypothesis. Does reduction of dietary fat reverse the altered astrocytic morphology remodeling via NF-κB suppression? The answers to this await further delineation. To establish a causal link between NF-κB signaling and the observed astrocytic morphological changes and behavioral alterations induced by HFD, future studies should employ astrocyte-specific NF-κB pathway inhibition. For instance, conditional knockout mice with targeted deletion of IKKβ or p65 (RelA) in astrocytes (e.g., using GFAP-Cre or Aldh1l1-CreERT2 driver lines) could be utilized to determine whether suppression of NF-κB activity mitigates HFD-induced astrocytic remodeling and depressive-like behaviors. Additionally, pharmacological inhibitors of NF-κB signaling administered selectively into the ventral hippocampus could complement genetic approaches and help delineate region-specific effects. These experiments would provide mechanistic insights into whether NF-κB acts as a critical mediator of diet-induced neuroinflammatory and behavioral phenotypes, and whether targeting this pathway could offer therapeutic potential in obesity-related neuropsychiatric conditions.

Accumulating evidence indicates that weight loss alone, independent of macronutrient composition, can alleviate depressive symptoms [38]. Obesity is associated with chronic low-grade systemic inflammation, insulin resistance, and hypothalamic-pituitary-adrenal (HPA) axis dysregulation—physiological abnormalities that are also implicated in the pathophysiology of depression. Caloric restriction and subsequent weight loss have been shown to reduce circulating proinflammatory cytokines, improve insulin signaling, and normalize HPA axis activity, which collectively may confer antidepressant effects. Additionally, weight reduction has been associated with increased brain-derived neurotrophic factor (BDNF) expression and improved neurogenesis, further supporting its potential role in mood regulation. Clinical and preclinical studies have reported improvements in affective symptoms following weight loss through behavioral, dietary, or bariatric interventions, regardless of macronutrient profile. Therefore, future research employing pair-fed or weight-matched control paradigms will be critical to delineate the relative contributions of dietary composition versus energy balance and weight reduction in mediating the observed neurobehavioral effects. Taken together, our findings suggest that dietary fat reduction may reverse HFD-induced astrocytic deficits by suppressing hippocampal NF-κB signaling, thereby mitigating neuroinflammatory responses and associated depressive-like behaviors.

## 5. Conclusions

In conclusion, our findings demonstrate that reduction of dietary fat is sufficient to improve HFD-induced metabolic disorder, depressive-like behavior, and ventral hippocampal astrocytic impairments in mice. These results suggested that dietary fat reduction may serve as a promising non-pharmacological intervention for individuals suffering from comorbid metabolic disorders and depression. Further research, particularly in human populations, is needed to evaluate its translational relevance and therapeutic applicability.

## Data Availability

The original contributions presented in this study are included in the article/Supplementary Material. Further inquiries can be directed to the corresponding author(s).

## Supplementary Materials

The following supporting information can be downloaded at: https://www.mdpi.com/article/10.3390/metabo15070485/s1, Supplementary Figure S1. Details of number of animals used in each experiment. Supplementary Figure S2. Effects of HFD on body weight, systemic glucose metabolism in mice. Supplementary Figure S3. Effects of HFD on exhibition of depression-like behaviors in mice. Supplementary Figure S4. Effects of the reduction of dietary fat on inflammatory factors in the hippocampus. Supplementary Figure S5. Effects of the reduction of dietary fat on astrocyte density in mice.
