# Supplementary material for: Reduction of Dietary Fat Rescues High-Fat Diet-Induced Depressive Phenotypes and the Associated Hippocampal Astrocytic Deficits in Mice
Source: Metabolites. 2025 Jul 18;15(7):485. doi: 10.3390/metabo15070485 (PMC12299380; doi:10.3390/metabo15070485)
Supplement: Supplementary file 1 [file metabolites-15-00485-s001.zip › Figure legends for suppl.pdf]

Figure legends for suppl. Figures

Figure 1. Details of number of animals used in each experiment. (a) Experimental procedures and number of animals used in each experiment of HFD study. **(b)** Experimental procedures and number of animals used in each experiment of change of the dietary composition study.

Figure 2. Effects of HFD on body weight, systemic glucose metabolism in mice. (a) Experimental timeline. **(b)** Quantitative results of body weight of mice during the feeding period. **(c)** Quantitative results of body weight of mice after the end of regimen **(d)** Quantitative results of energy intake of mice. **(e)** Alterations of blood glucose levels of mice in the IPGTT were shown in left panel and the quantitative results of area under curves (AUC) were shown in the right panel. **(f)** Alterations of blood glucose levels of mice in the IPITT were shown in left panel and the quantitative results of AUC were shown in the right panel. **(g)** Quantitative results of fasting plasma levels of glucose in mice. **(h)** Quantitative results of fasting plasma levels of insulin in mice. **(i)** Quantitative results of HOMA-IR index. $^{*}p < 0.05$, $^{**}p < 0.01$, $^{***}p < 0.001$, $^{****}p < 0.0001$, vs. SD, unpaired Student's *t* test in scatter plots and repeated measure two-way ANOVA in line graphs. Numbers given in the parentheses indicates the sample sizes. Values represent mean ± S.E.M. from three independent experiments.

Figure 3. Effects of HFD on exhibition of depression-like behaviors in mice. (a) Quantitative results of SPT. **(b)** Quantitative results of FST. **(c)** Quantitative results of TST. *p < 0.05, **p < 0.01, ***p < 0.001, ****p < 0.0001, vs. SD, unpaired Student's t test in scatter plots and repeated measure two-way ANOVA in line graphs. Numbers given in the parentheses indicates the sample sizes. Values represent mean ± S.E.M. from three independent experiments.

Figure 4. Effects of the reduction of dietary fat on inflammatory factors in the hippocampus. Protein level of TNF-α, IL-1β, and IL-6 was examined by ELISA. Each value represents mean±SEM from at least 10 mice in each group. *P < 0.05, **P < 0.01, ***P < 0.001, ****P < 0.0001, vs. respective SD/SD group. #p < 0.05, ##p < 0.01, ###p < 0.001, ####p < 0.0001 vs. respective HFD/HFD group.

Figure 5. Effects of the reduction of dietary fat on astrocyte density in mice. Quantification of % area stained with GFAP (n=10) in each group. Values shown in graphs represent the mean value ± SEM.
