# Supplementary material for: Reduction of Dietary Fat Rescues High-Fat Diet-Induced Depressive Phenotypes and the Associated Hippocampal Astrocytic Deficits in Mice
Source: Metabolites. 2025 Jul 18;15(7):485. doi: 10.3390/metabo15070485 (PMC12299380; doi:10.3390/metabo15070485)
Supplement: Supplementary file 1 [file metabolites-15-00485-s001.zip › Suppl. Figure S1.pdf]

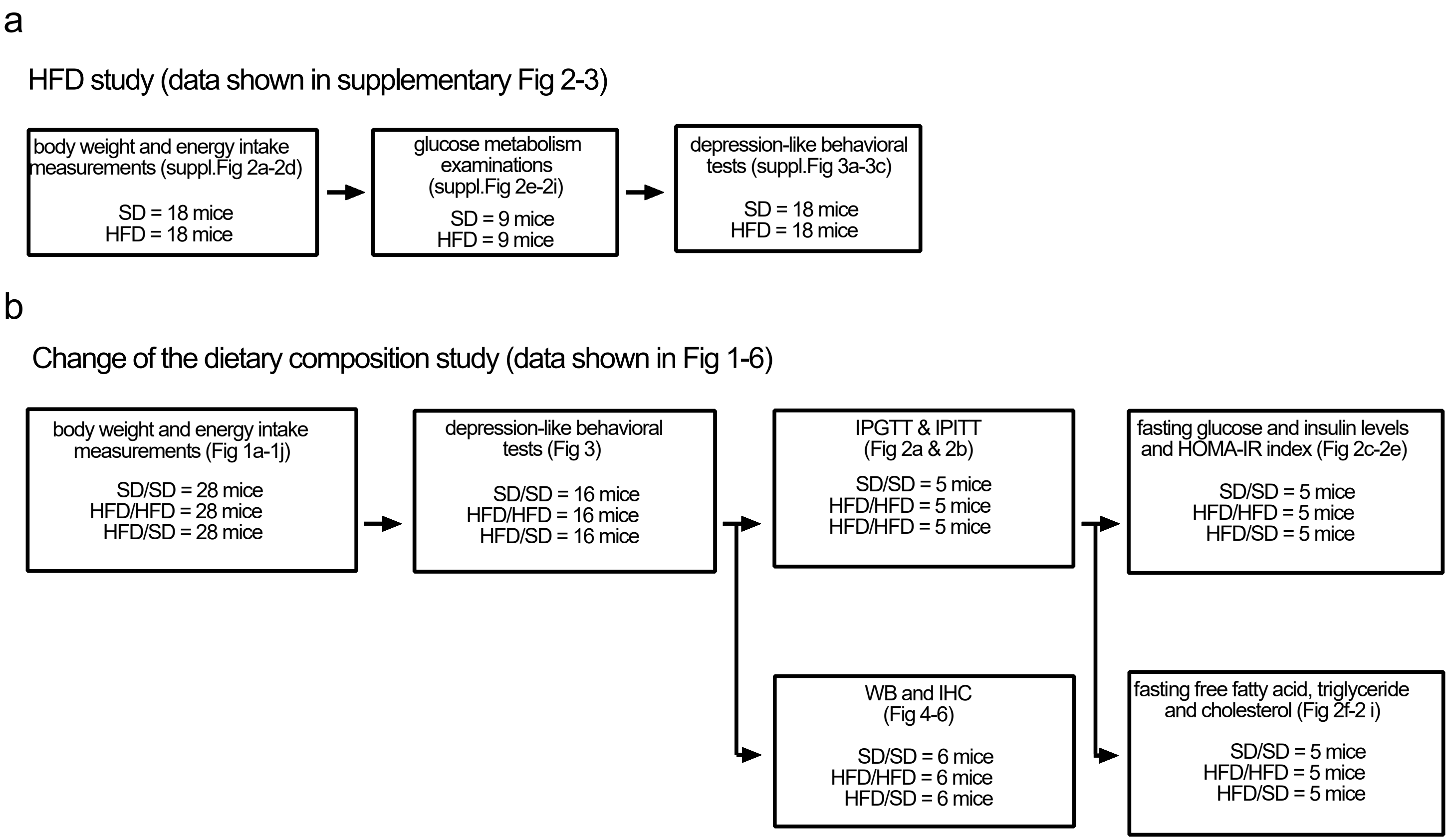
a
HFD study (data shown in supplementary Fig 2-3)
body weight and energy intake measurements (suppl.Fig 2a-2d)
SD = 18 mice
HFD = 18 mice
glucose metabolism examinations (suppl.Fig 2e-2i)
SD = 9 mice
HFD = 9 mice
depression-like behavioral tests (suppl.Fig 3a-3c)
SD = 18 mice
HFD = 18 mice
b
Change of the dietary composition study (data shown in Fig 1-6)
body weight and energy intake measurements (Fig 1a-1j)
SD/SD = 28 mice
HFD/HFD = 28 mice
HFD/SD = 28 mice
depression-like behavioral tests (Fig 3)
SD/SD = 16 mice
HFD/HFD = 16 mice
HFD/SD = 16 mice
IPGTT & IPITT (Fig 2a & 2b)
SD/SD = 5 mice
HFD/HFD = 5 mice
HFD/HFD = 5 mice
WB and IHC (Fig 4-6)
SD/SD = 6 mice
HFD/HFD = 6 mice
HFD/SD = 6 mice
fasting glucose and insulin levels and HOMA-IR index (Fig 2c-2e)
SD/SD = 5 mice
HFD/HFD = 5 mice
HFD/SD = 5 mice
fasting free fatty acid, triglyceride and cholesterol (Fig 2f-2 i)
SD/SD = 5 mice
HFD/HFD = 5 mice
HFD/SD = 5 mice
