# Supplementary material for: Reduction of Dietary Fat Rescues High-Fat Diet-Induced Depressive Phenotypes and the Associated Hippocampal Astrocytic Deficits in Mice
Source: Metabolites. 2025 Jul 18;15(7):485. doi: 10.3390/metabo15070485 (PMC12299380; doi:10.3390/metabo15070485)
Supplement: Supplementary file 1 [file metabolites-15-00485-s001.zip › supplementary information.pdf]

Reduction of dietary fat rescues high-fat diet-induced depressive phenotypes and the associated hippocampal astrocytic deficits in mice

†Kai-Pi Cheng [1], †Hsin-Hao Chao[2], Chin-Ju, Hsu[3], Sheng-Fang Tsai[4,5], Yen-Ju Chiu[3], Yun-Min Kuo[4,5], Yun-Wen Chen[3*]

[1]Department of Internal Medicine, National Cheng Kung University Hospital, College of Medicine, National Cheng Kung University, Tainan, Taiwan.

[2]Department Of Psychiatry, Ditmanson Medical Foundation Chiayi Christian Hospital, Chiayi, Taiwan

[3]Department of Pharmacology, College of Medicine, National Cheng Kung University, Tainan, Taiwan

[4]Institute of Basic Medical Sciences, College of Medicine, National Cheng Kung University, Tainan, 70101, Taiwan

[5]Department of Cell Biology and Anatomy, College of Medicine, National Cheng Kung University, Tainan, 70101, Taiwan

† These authors contributed equally

Supplementary Information

Supplementary Figure S1. Details of number of animals used in each experiment.
Supplementary Figure S2. Effects of HFD on body weight, systemic glucose metabolism in mice.
Supplementary Figure S3. Effects of HFD on exhibition of depression-like behaviors in mice.
Supplementary Figure S4. Effects of the reduction of dietary fat on inflammatory factors in the hippocampus.
Supplementary Figure S5. Effects of the reduction of dietary fat on astrocyte density in mice.

Figure legends of supplementary figures
