# Supplementary material for: Reduction of Dietary Fat Rescues High-Fat Diet-Induced Depressive Phenotypes and the Associated Hippocampal Astrocytic Deficits in Mice
Source: Metabolites. 2025 Jul 18;15(7):485. doi: 10.3390/metabo15070485 (PMC12299380; doi:10.3390/metabo15070485)
Supplement: Supplementary file 1 [file metabolites-15-00485-s001.zip › WB for review (final).pdf]

Figure 4(a)

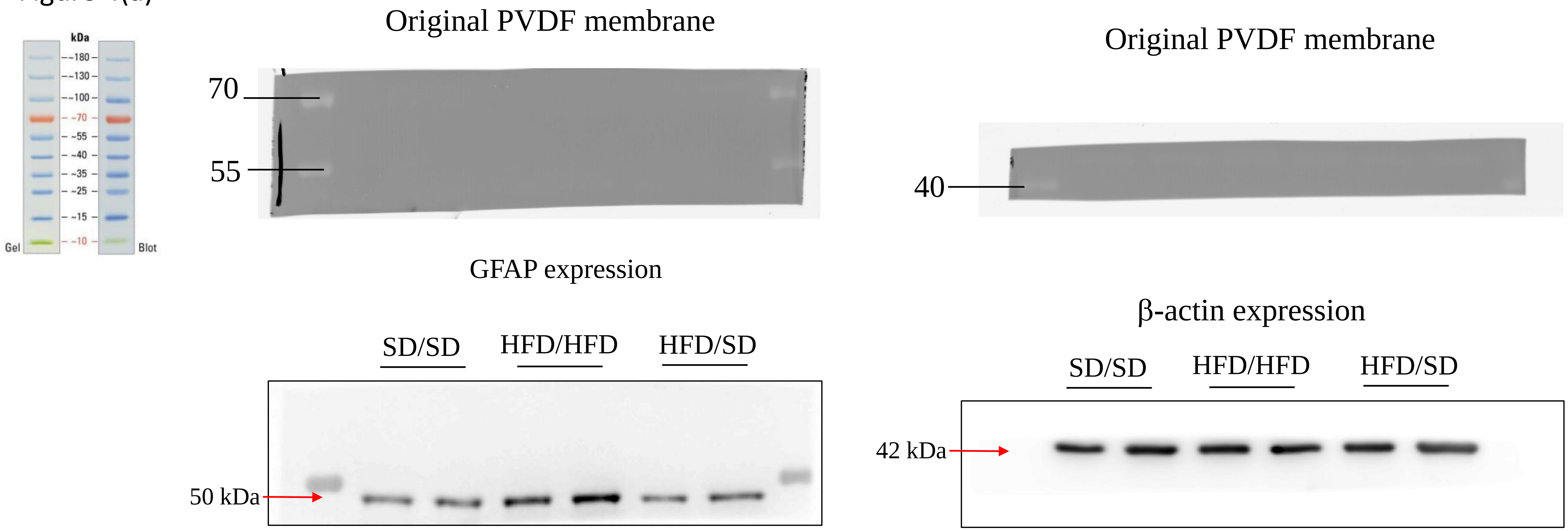
kDa
~180
~130
~100
~70
~55
~40
~35
~25
~15
~10
Gel
Blot
Original PVDF membrane
70
55
GFAP expression
SD/SD
HFD/HFD
HFD/SD
50 kDa
Original PVDF membrane
40
β-actin expression
SD/SD
HFD/HFD
HFD/SD
42 kDa

Figure 5(a)

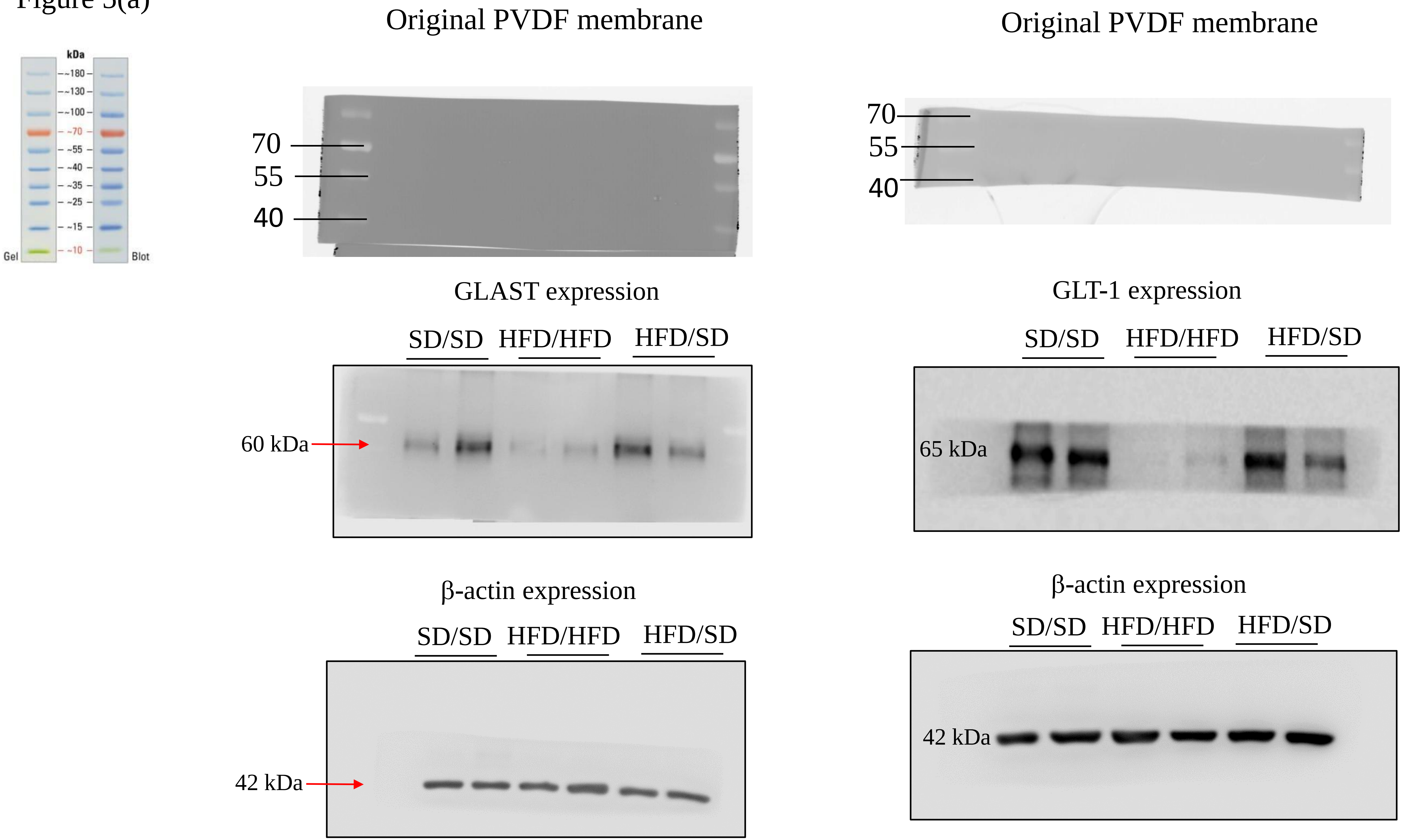
Original PVDF membrane
Original PVDF membrane
kDa
~180
~130
~100
~70
~55
~40
~35
~25
~15
~10
Gel
Blot
70
55
40
70
55
40
GLAST expression
GLT-1 expression
SD/SD
HFD/HFD
HFD/SD
60 kDa
65 kDa
β-actin expression
β-actin expression
SD/SD
HFD/HFD
HFD/SD
SD/SD
HFD/HFD
HFD/SD
42 kDa
42 kDa

Figure 6 (a)

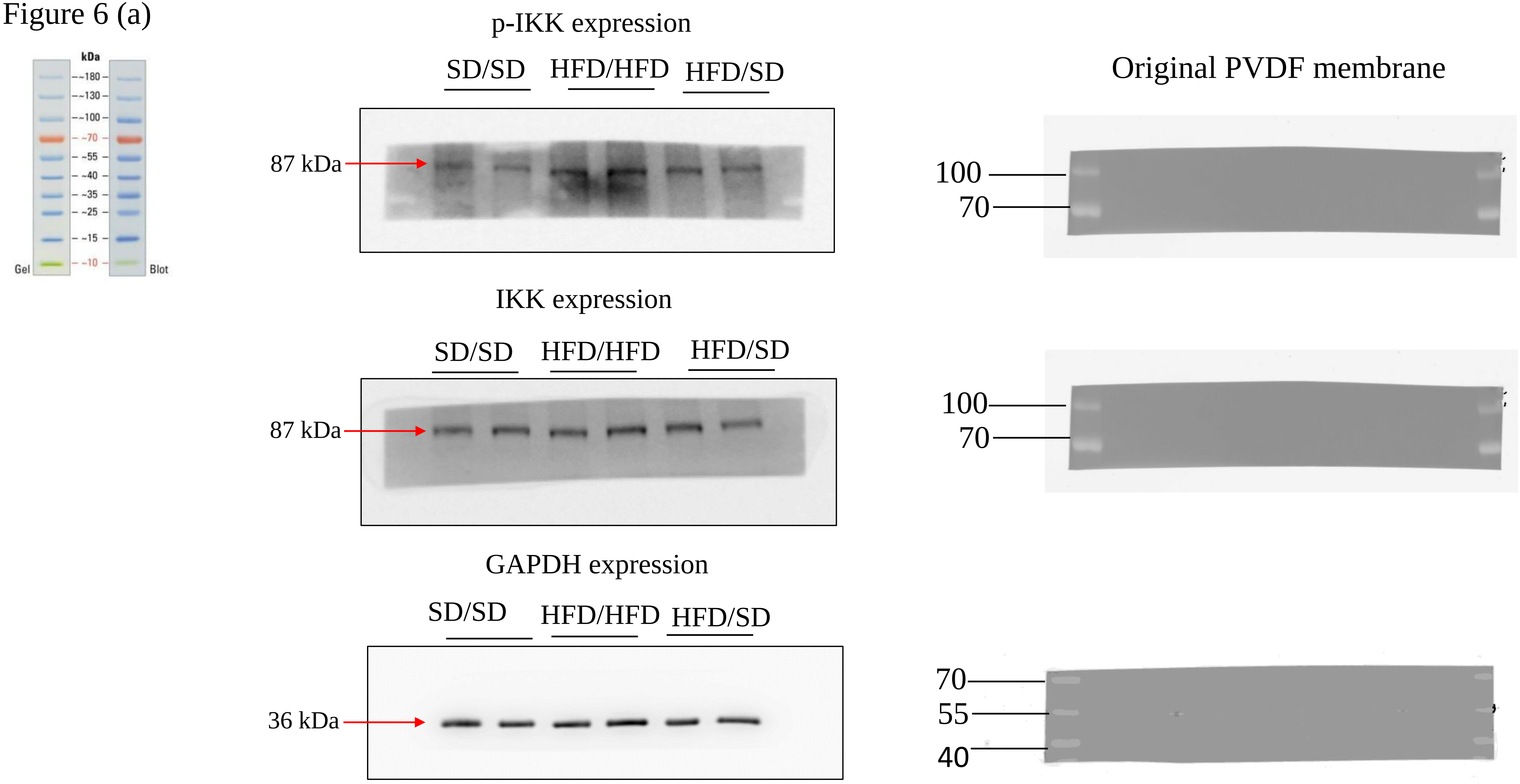
kDa
~180
~130
~100
~70
~55
~40
~35
~25
~15
~10
Gel
Blot
p-IKK expression
SD/SD
HFD/HFD
HFD/SD
87 kDa
IKK expression
SD/SD
HFD/HFD
HFD/SD
87 kDa
GAPDH expression
SD/SD
HFD/HFD
HFD/SD
36 kDa
Original PVDF membrane
100
70
100
70
70
55
40
