# Supplementary figures and images for: Reduction of Dietary Fat Rescues High-Fat Diet-Induced Depressive Phenotypes and the Associated Hippocampal Astrocytic Deficits in Mice
Source: Metabolites. 2025 Jul 18;15(7):485. doi: 10.3390/metabo15070485 (PMC12299380; doi:10.3390/metabo15070485)

**a**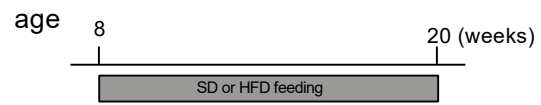**b**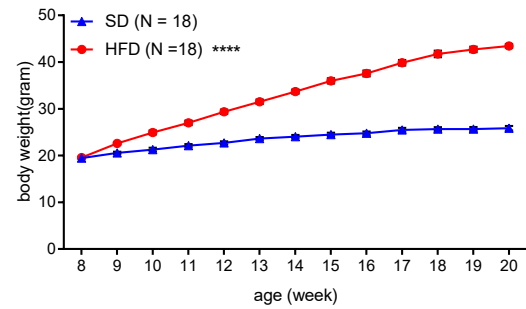**c**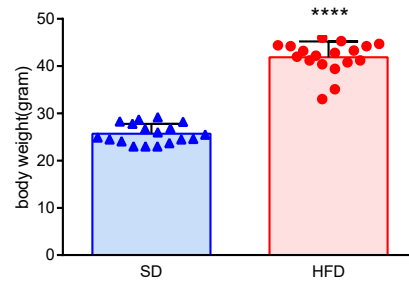**d**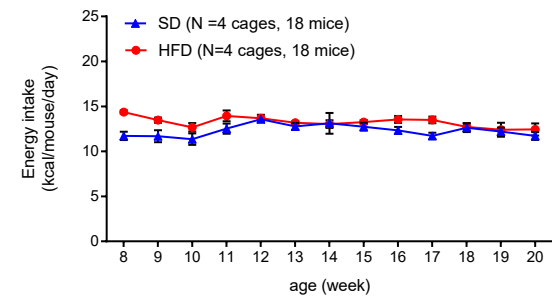**e**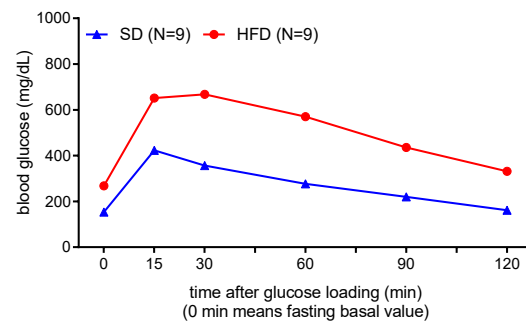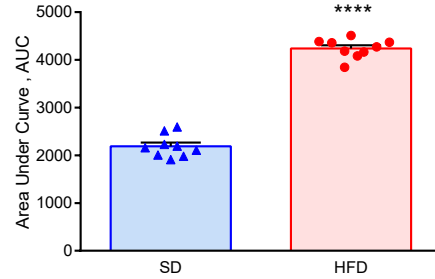**f**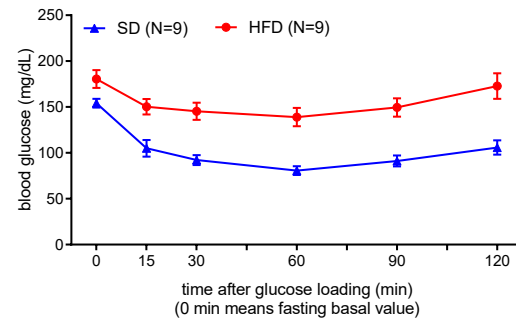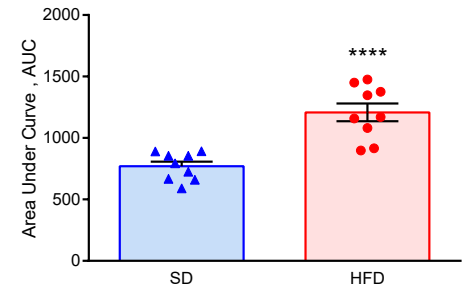**g**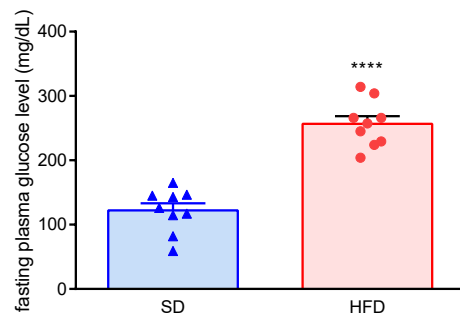**h**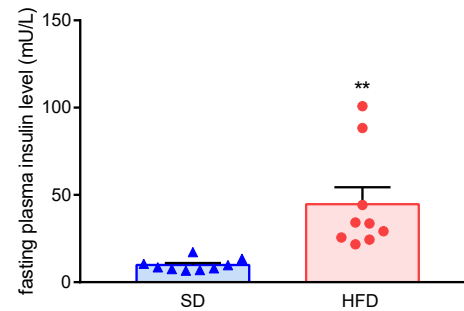**i**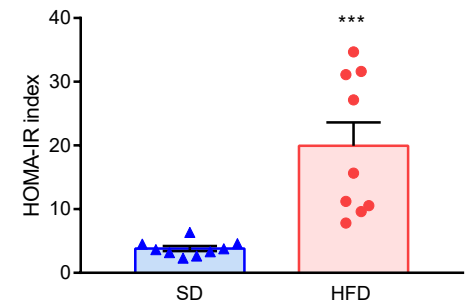

Supplement: Supplementary file 1 [file metabolites-15-00485-s001.zip › Suppl. Figure S2.pdf]

sucrose preference test

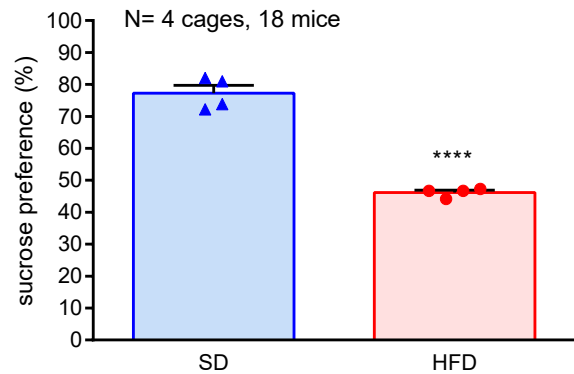

forced swimming test

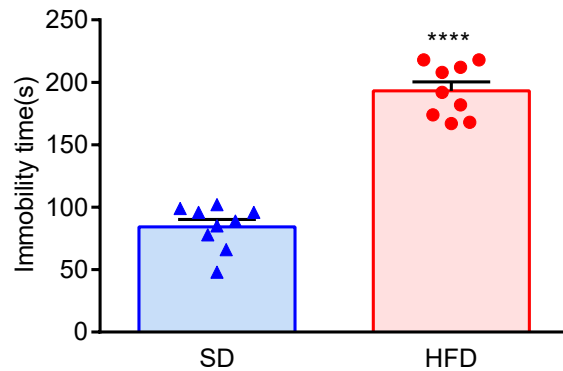

tail suspension test

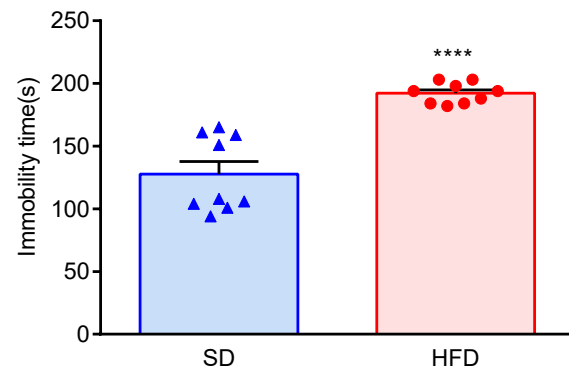

Supplement: Supplementary file 1 [file metabolites-15-00485-s001.zip › Suppl. Figure S3.pdf]

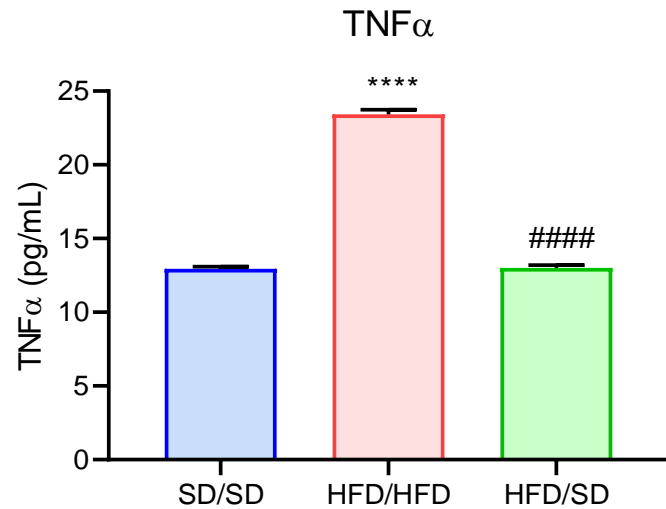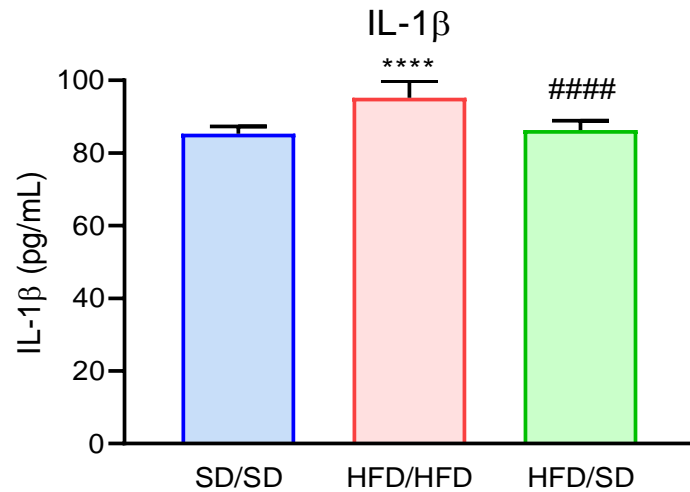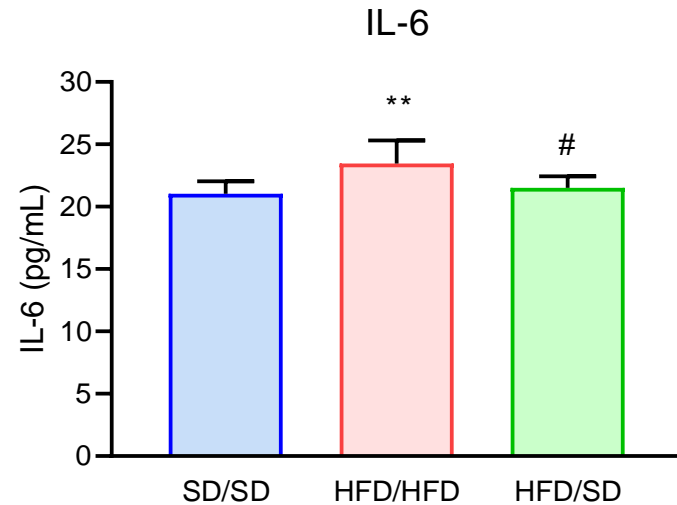

Supplement: Supplementary file 1 [file metabolites-15-00485-s001.zip › Suppl. Figure S4.pdf]

# astrocyte density

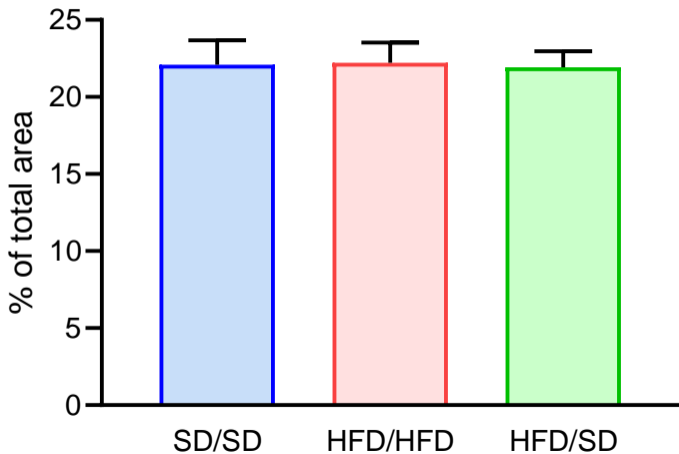

Supplement: Supplementary file 1 [file metabolites-15-00485-s001.zip › Suppl. Figure S5.pdf]
